# Supplementary material for: Gold Nanodisks Plasmonic Array for Hydrogen Sensing at Low Temperature
Source: Sensors (Basel). 2019 Feb 5;19(3):647. doi: 10.3390/s19030647 (PMC6387255; doi:10.3390/s19030647)
Supplement: Supplementary file 1 [file sensors-19-00647-s001.pdf]

Supplementary Materials

Gold nanodisks plasmonic array for hydrogen sensing at low temperature

Marco Sturaro, Gabriele Zacco, Pierfrancesco Zilio, Alessandro Surpi, Marco Bazzan, Alessandro Martucci*

Finite element simulations

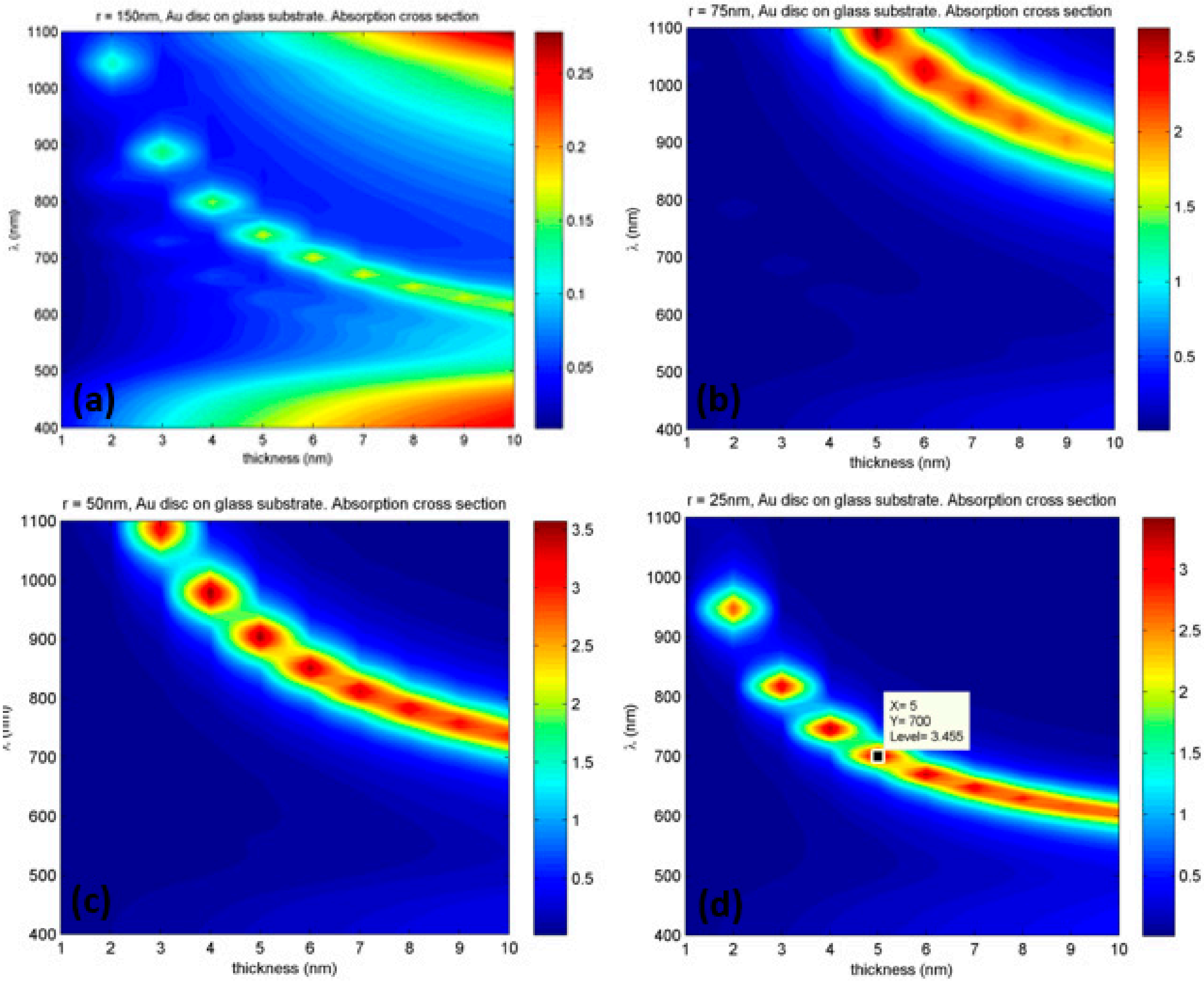

Figure S1 Absorption cross-section of a single Au ND as a function of wavelength and ND thickness for radius r = 150 nm (a), 75 nm (b), 50 nm (c), 25 nm (d)

Figure S1 shows the wavelength dependence of absorption cross-section of a single Au ND with different thickness, from 1 to 10 nm, and different radius, from 150 nm to 25 nm. Smaller and thicker NDs have LSPR centered at shorter wavelengths. Optimum thickness was found to be 5 nm for all the investigated diameters. In Figure S1(a) it is shown the cross-

section of the ND absorption with 150 nm radius. The resonance presents three nodes, probably due to higher harmonic of bipolar resonances that appears in the upper right of the graph. For ND thickness of 7-8 nm, the cross-section has a small peak at $\lambda \approx 650$ nm. In any case the intensity of the LSPR is relatively low in all considered ranges.
In Figure S1(b) it is presented the absorption cross-section for ND with radius of 75 nm. The absorption has a very intense band (one order of magnitude higher in absorption compared to ND with a radius of 150 nm) and the maximum is around the wavelength of 1100 nm for a height of 5 nm. For ND radius of 50 nm with thickness of 5 nm, the maximum absorption is slightly blue-shifted as shown in Figure S1(c), at $\lambda \approx 850$ nm.
Finally, with a ND radius of 25 nm, the maximum absorption wavelength enters in the visible range, at 700 nm, with an optimum height at 5 nm as shown in Figure S1 (d).

SEM characterizations

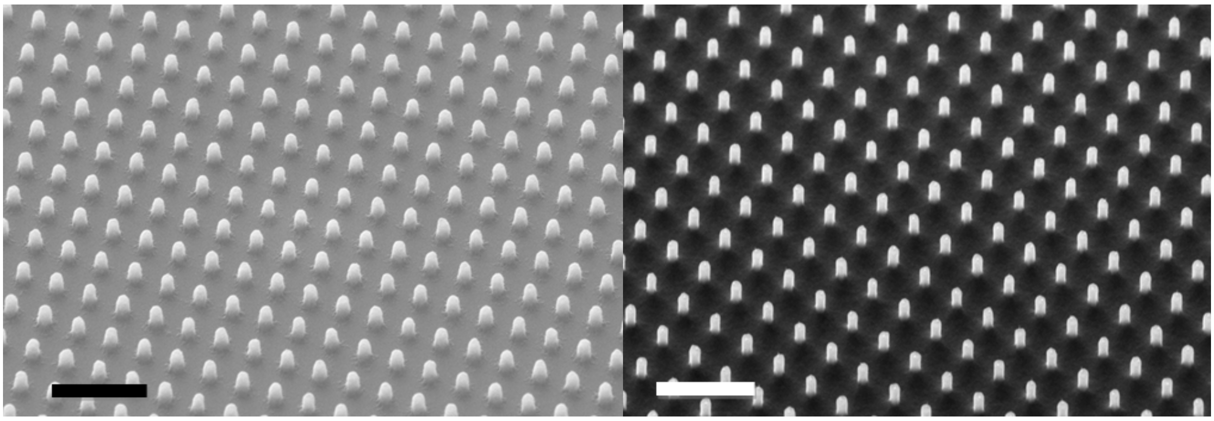

Figure S2 SEM images of masters used for samples with NDs diameters of 150 nm (left) and 50 nm (right). Scale bars are 1 μm for both images.

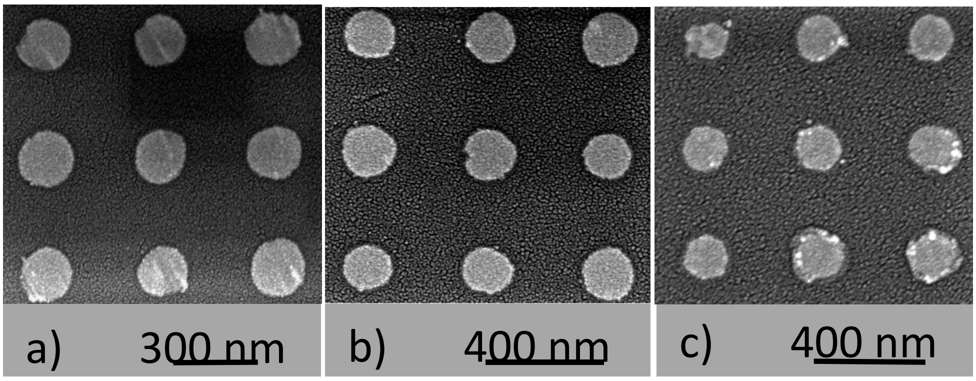

Figure S3 SEM images of the Au NDs array after (a) 30 °C, (b) 150 °C, (c) 250 °C stabilization in air for 1 hour. The mean diameter with the relative standard deviation are 166±10 nm, 152±9 nm, 143±11 nm, respectively.

Optical absorbance

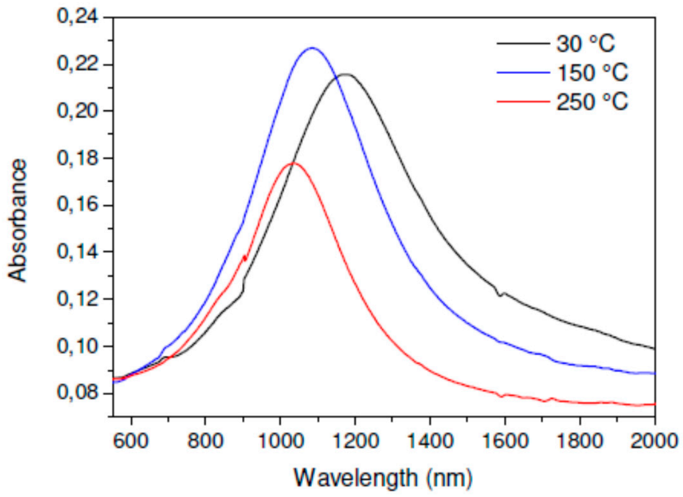

Figure S4 Absorbance spectra of the Au NDs after stabilization at 30 °C, 150 °C and 250 °C in air for 1 hour.

Ellipsometry measurements

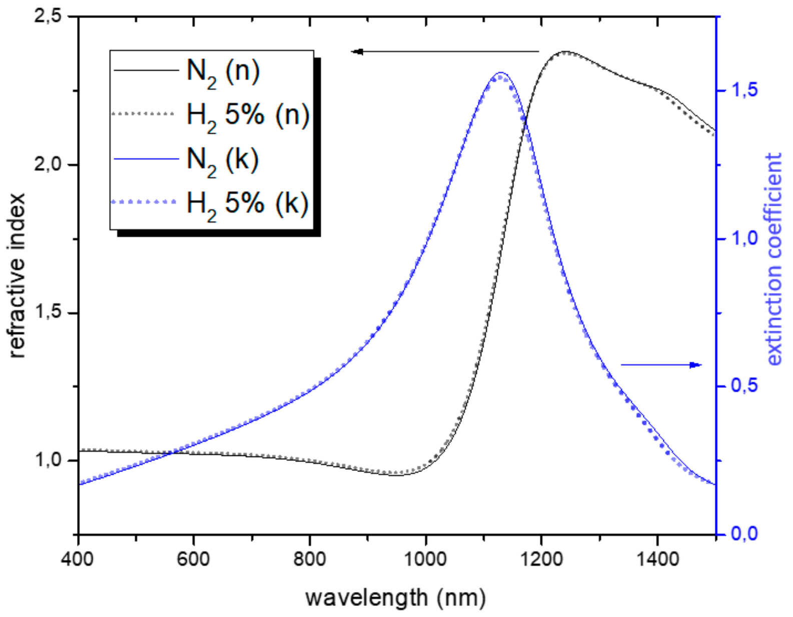

Figure S5 Refractive index (n) and extinction coefficient (k), respectively plotted in black and blue, for Au NDs in presence of inert atmosphere (nitrogen, solid curves) or hydrogen-rich atmosphere (5% vol H_2 in Argon, dashed curves).

XRD measurements

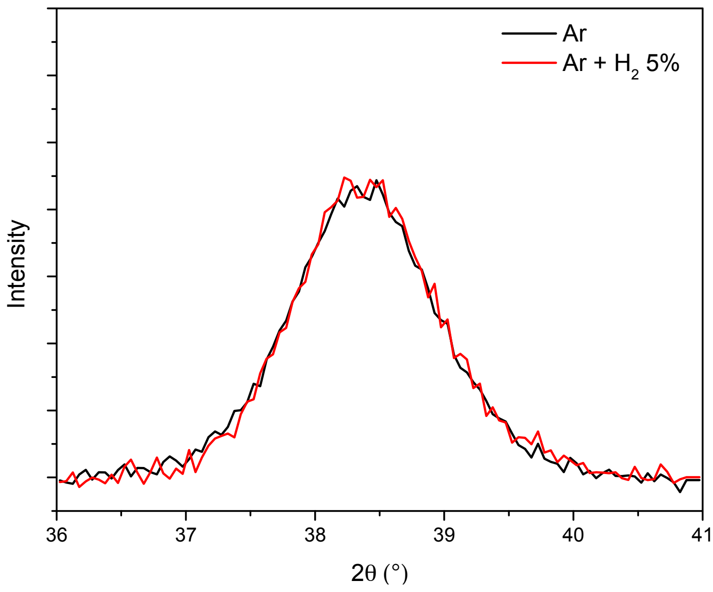

Figure S6 XRD patterns for the (111) Bragg peak of Au NDs in presence of inert atmosphere (Argon, black curve) or hydrogen-rich atmosphere (5% vol H_2 in Argon, red curve).

XRD measurements performed on Au NDs sample confirmed the presence of crystalline Au. Crystallite mean size was evaluated through Sherrer equation to be 6 ± 0.5 nm. To trace the structural changes of the Au NDs, a sample was loaded into the cell under a flow of 5 vol.% H_2/Ar at 0.4L/min, during which the XRD patterns were recorded using a continuous mode in the 36° to 41° 2θ range, where is located the Au main diffraction peak for (111) planes at 38.3°. No changes were detected in shape and position of the peak during gas exposure.
